# Perivascular-Like Cells Contribute to the Stability of the Vascular Network of Osteogenic Tissue Formed from Cell Sheet-Based Constructs

**DOI:** 10.1371/journal.pone.0041051

**Published:** 2012-07-19

**Authors:** Luís F. Mendes, Rogério P. Pirraco, Wojciech Szymczyk, Ana M. Frias, Tírcia C. Santos, Rui L. Reis, Alexandra P. Marques

**Affiliations:** 1 3B’s Research Group–Biomaterials, Biodegradables and Biomimetics, University of Minho, Guimarães, Portugal; 2 ICVS/3B’s, PT Government Associate Laboratory, University of Minho, Braga/Guimarães, Portugal; University of Minnesota Medical School, United States of America

## Abstract

In recent years several studies have been supporting the existence of a close relationship in terms of function and progeny between Mesenchymal Stem Cells (MSCs) and Pericytes. This concept has opened new perspectives for the application of MSCs in Tissue Engineering (TE), with special interest for the pre-vascularization of cell dense constructs. In this work, cell sheet technology was used to create a scaffold-free construct composed of osteogenic, endothelial and perivascular-like (CD146^+^) cells for improved in vivo vessel formation, maturation and stability. The CD146 pericyte-associated phenotype was induced from human bone marrow mesenchymal stem cells (hBMSCs) by the supplementation of standard culture medium with TGF-β1. Co-cultured cell sheets were obtained by culturing perivascular-like (CD146^+^) cells and human umbilical vein endothelial cells (HUVECs) on an hBMSCs monolayer maintained in osteogenic medium for 7 days. The perivascular-like (CD146^+^) cells and the HUVECs migrated and organized over the collagen-rich osteogenic cell sheet, suggesting the existence of cross-talk involving the co-cultured cell types. Furthermore the presence of that particular ECM produced by the osteoblastic cells was shown to be the key regulator for the singular observed organization. The osteogenic and angiogenic character of the proposed constructs was assessed *in vivo*. Immunohistochemistry analysis of the explants revealed the integration of HUVECs with the host vasculature as well as the osteogenic potential of the created construct, by the expression of osteocalcin. Additionally, the analysis of the diameter of human CD146 positive blood vessels showed a higher mean vessel diameter for the co-cultured cell sheet condition, reinforcing the advantage of the proposed model regarding blood vessels maturation and stability and for the *in vitro* pre-vascularization of TE constructs.

## Introduction

The interest on the cell sheet engineering concept for regenerative medicine purposes has been increasing over the years. Gradually, this approach is being established as a reliable alternative for traditional tissue engineering (TE) and regenerative medicine methods, namely the use of biodegradable scaffolds to create tissue substitutes and the injection of isolated cells [1]. The revolutionary concept consisted on the use of poly(N-isopropylacrylamide) (PIPAAm), to produce thermoresponsive culture surfaces that allow cells recovery, within their own extracellular matrix (ECM), as a sheet with cohesive cell-cell and cell-ECM interactions [2]. For the past 10 years, several works have shown the potential of this technology for cornea [3] and myocardial tissues reconstitution [4], hepatocyte transplantation [5], renal tube epithelial cell transfer [6] and for bone tissue engineering applications [7]. Moreover, several reports have also proved the advantages of cell sheets stacking and of patterned thermoresponsive surfaces to obtain co-cultured cell sheets [8]–[11], to further enhance the similarities of the created constructs with *in vivo* tissues. At the same time, the limited and non-functional vascularization of thick cell sheet-based tissue engineering constructs after implantation has been tackled by co-culturing endothelial cells with other progenitor or committed cells. In fact, like for traditional TE strategies, the pre-vascularization of cell sheets-based constructs has been proposed as a way to circumvent this problem [8], [9]. According to Rouwkema and colleagues [12], the pre-vascularization strategy can dramatically reduce, in comparison to approaches that depend on scaffold design and angiogenic factors delivery, the time needed to vascularize the implant.

Despite endothelial cells being the cells that line the blood vessels of the entire cardiovascular system [13], perivascular cells, specially pericytes, were shown to have great impact over vascularization, contributing to blood vessel stability and maturation and for the regulation of microvascular blood flow [14]–[16]. In addition, stable vessels with lowest turnover rates, such as in brain and retina, are proved to have highest density of pericytes [17]. Thus, the use of endothelial cells for the successful generation of TE constructs is still dependent on the recruitment of host mural cells to new formed vasculature, leading the transition from a growing vascular network to a quiescent vascular phenotype [18], [19]. Recent findings in this field have catapulted the number of works exploring pericytes progeny and multipotency, as well as their significance for the advancement of the TE field [20]–[23]. Pericytes establish important direct cell-cell contact with endothelial cells of immature blood vessels [19] and some studies have suggested that, *in vivo,* pericytes may serve as guiding structures aiding outgrowth of endothelial cells to form early capillary sprouts [24], [25]. Co-expression of several surface markers between pericytes and mesenchymal stem cells (MSCs), as well as a mesenchymal/fibroblast-like morphology, led Caplan to suggest in 2008 [26] that all the MSCs are pericytes and later [27] that for almost every blood vessel in the body a perivascular cell niche of MSCs should exist. CD146, a surface marker co-expressed by a subpopulation of hBMSCs and by some populations of pericytes [28], is an important adhesion molecule for vascular endothelial cell activity and angiogenesis [29]. This transmembrane glycoprotein has important functions in early and late development and it has been suggested to play an important role in cancer, angiogenesis, cardiovascular diseases and placentation [30]. Moreover, a significant number of studies in the field of cancer research have assigned to CD146 a critical role in tumor growth and metastasis, as well as in tumor angiogenesis [31], [32]. At the core of an efficient cross-talk between endothelial cells, mural cells and ECM, are thought to be a diversity of proteins, namely PDGF, TGF, VEGF, Collagen, laminin and others, both soluble or trapped on the ECM [33]–[35]. Between them, while PDGF and TGF have an undeniable and well-studied contribution to the pericitic-endothelial cells interactions and vessel stabilization [33], [36], the function of VEGF seems to be more controversial because while it mainly stimulates EC proliferation and migration also seems to ablate pericyte coverage of nascent vascular sprouts [37]. Interestingly, type- I collagen and laminin-1 are referred to have positive and negative influence, respectively, over capillary morphogenesis *in vitro* [*35*].

The main goal of this work was to develop a three-dimensional osteogenic cell dense construct combining endothelial and perivascular-like cells differentiated from hBMSCs, as a way to accelerate the vascularization of the engineered construct *in vivo* and thus contribute to its survival. We hypothesized that the incorporation of perivascular-like (CD146^+^) cells, directly interacting with endothelial cells, could further enhance the effect of the pre-vascularization by promoting the maturation and stabilization of the newly formed vasculature. To verify our assumptions a co-culture system was created *in vitro*, by culturing HUVECs and induced perivascular-like (CD146^+^) cells on a confluent layer of hBMSCs-derived osteogenic cells. The *in vivo* transplantation of the co-cultured constructs combining two osteogenic cell sheets with HUVECs and perivascular-like (CD146^+^) cells in between permitted to demonstrate the active role of these cells in the formation of the new vasculature as well as its influence over its maturation and stability as shown by the increased number and blood vessel diameter at early time points. While these findings and the osteogenic character of the created constructs demonstrate their potential for bone tissue engineering purposes it is our believe that it might be also considered as a suitable model for the *in vitro* pre-vascularization of TE constructs.

## Results

### TGF-β1 Induces the Expression of CD146 Molecule and Causes Cellular Morphological Changes

The expression pattern of some surface markers was followed, on hBMSCs cultured in multiple conditions, by flow cytometry and immunocytochemistry using several antibodies.

**Figure 1:**
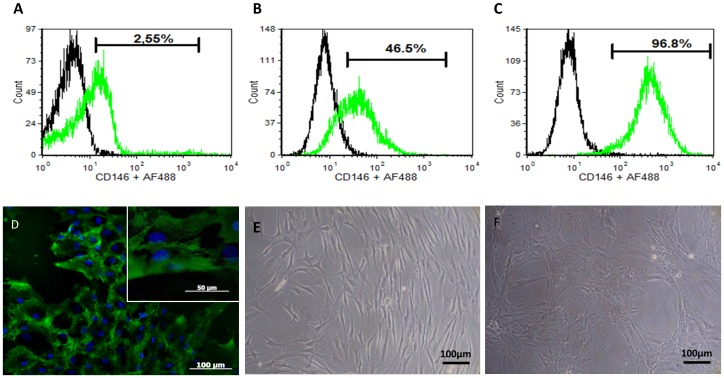
Representative flow cytometry and immunocytochemistry analysis of human bone marrow derived cells at different passages and cultured with and without TGF-β1. (A) CD146 expression of bone marrow mononuclear fraction at isolation day; (B) CD146 expression on hBMSCs (P5) cultured in complete α-MEM; (C; D) CD146 expression analysis, by flow cytometry (C) and immunocytochemistry (green) (D), on hBMSCs (P5) cultured in complete α-MEM supplemented with 1 ng/mL TGF-β1 for 7 days; Evolution of cell morphology of hBMSCs (E) before and (F) after culture in α-MEM +1 ng/mL TGF-β1 for 7 days. For immunocytochemistry DAPI (blue) was used as nuclear staining. Right upper corner image in D represent a higher magnification.

As previously described by others [28], [38], we identified the presence of CD146^+^ cells within the mononuclear fraction of human bone marrow aspirates. Flow cytometry performed on the mononuclear fraction from marrow at the isolation day, revealed the presence of a small CD146^+^ sub-population representing approximately 2.5% of the total cellular content (Figure 1A). The frequency of cell markers, such as CD105, CD73 and CD90 normally associated to the mesenchymal phenotype was less than 2% in the mononuclear fraction of the marrow (Figure S1). However, after selection by adhesion to TCPS these values increased and were kept stable along different passages. For a representative sample (P5) cultured in complete α-MEM, the percentage of CD146^+^ cells was approximately 46% (Figure 1B), and more than 98% of the hBMSCs expressed the surface markers CD105, CD73 and CD90 (Figure S1).

In what concerns the effect of the TGF-β1 over the hBMSCs surface markers expression, the number of cells expressing CD146 antigen, as well as the amount of CD146 antigen, increased after the treatment. TGF-β1 is associated with the induction of a mature smooth muscle phenotype in 10T1/2 cells [36], capable of stimulating the NG2 pericyte associated marker expression in mouse embryo fibroblasts [34] and the expression of contractile proteins in cultured vSMC [39] while it is also referred as a potential growth inhibitor [23]. In a representative population of hBMSCs (P5), cultured for 7 days in α-MEM supplemented with 1 ng/mL TGF-β1, more than 97% of the analyzed cells were positive for CD146 (Figure 1C), and maintained the expression of CD105, CD73 and CD90 (≥98%) (Figure S1). The Immunocytochemistry analysis of the monocultures of the derived CD146^+^ cells after TGF-β1 treatment showed the ubiquity of the CD146 antigen over their surface confirming the flow cytometry results, as well as their characteristic “star morphology” with extended processes between neighboring cells (Figure 1D). This pronounced morphological change after culture with TFG-β1 (CD146^+^ cells) was also clearly observed by contrast phase microscopy in comparison to hBMSCs (Figure 1 E;F).

### Osteogenic Cells Derived from hBMSCs Support Endothelial and Perivascular-like (CD146^+^) Cells Adhesion and Survival Allowing the Fabrication of a Cell Sheet-Based Construct

**Figure 2:**
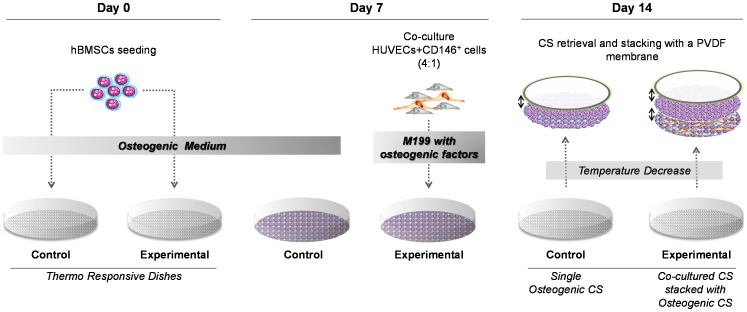
*In vitro* culture methodology to obtain a stacked co-cultured cell sheets (CS)-based model. hBMSCs were seeded and cultured for 7 days in osteogenic medium in thermoresponsive dishes. To obtain co-cultured CS, HUVECs and perivascular-like (CD146^+^) cells were cultured, at a ratio of 4∶1, on the osteogenic CS in M199 supplemented with osteogenic factors for further 7 days (experimental). Control homotypic osteogenic CS were maintained in osteogenic medium. At day 14, CS were retrieved from the thermoresponsive dishes by temperature decrease and the experimental model was built by stacking of a homotypic osteogenic CS onto the co-cultured CS using a poly(vinylidene diﬂuoride) (PVDF) membrane.

**Figure 3:**
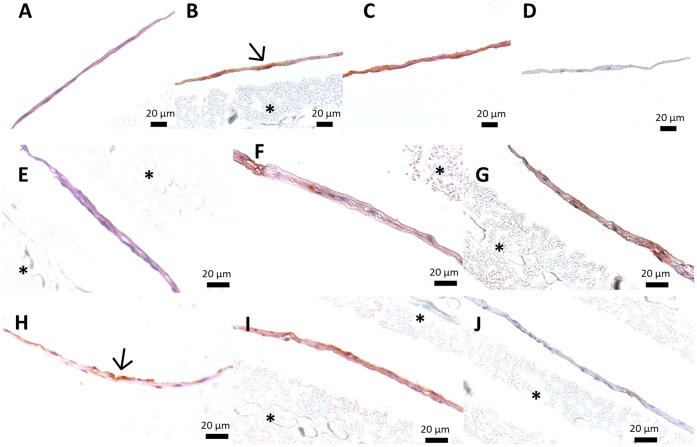
Histological characterization of single and co-cultured cell sheets after 14 days in culture in osteogenic medium and after detachment by temperature decrease and contraction. Single osteogenic cell sheet derived from hBMSCs A) after H&E staining and immunostained for (B) osteocalcin and (C) type-I collagen; Co-cultured cell sheets after (E) H&E staining and immunostaining for (F) CD31, (G) CD146, (H) osteocalcin and (I) Type-I collagen. Identification of positive signal was determined in comparison to immunocytochemistry negative controls (D;J). * PVDF membrane used to protect cell sheet during processing.

The basis of our concept relies on the production of a scaffold-free construct with osteogenic capacity and suitable properties for endothelial cell survival, migration and interaction with perivascular-like (CD146^+^) cells. This approach is essentially dependent on the fabrication of cells sheets by optimizing ECM production accordingly to the type of cells used and the envisaged application. In this work, hBMSCs were cultured for 14 days on PIPAAm thermoresponsive surfaces (Figure 2) in osteogenic medium with high concentrations of ascorbic acid in order to obtain osteogenic cell sheets. After retrieval by temperature decrease (Figure S2), the nature of the produced cell sheets was analyzed by H&E staining and by immunohistochemistry for osteocalcin and type-I collagen deposition. H&E performed on histological sections showed that hBMSCs form thin layers of contiguous cells embedded in a matrix (Figure 3A). Additionally, the positive staining for osteocalcin, a protein produced by mature osteoblasts during mineralization (Figure 3B), and for type-I collagen (Figure 3C), the most abundant protein in the organic bone matrix synthesized by active osteoblasts [40], demonstrated the deposition of ECM characteristic of the commitment of hBMSCs towards the osteogenic lineage. Endothelial and perivascular-like (CD146^+^) cells were cultured on the osteogenic cells at day 7 and maintained for the total 14 days of culture in order to create co-cultured osteogenic cell sheets. Histologically, the co-cultured cell sheets showed a cellular organization similar to single osteogenic cell sheets but with increased thickness and overlapped cells corresponding to colonies of endothelial and CD146^+^ cells (Figure 3E). These results were confirmed by the immunolocalization of CD31 (Figure 3F) and CD146 (Figure 3G) positive cells. As for the single osteogenic cell sheets, the deposition of osteocalcin (Figure 3H) and type-I collagen (Figure 3I) attested the osteogenic commitment of the *in vitro* system. Both on single and co-cultured cell sheets it was clear some intense areas of osteocalcin staining (Arrow on figure 3B;H), which seems to indicate unequal distribution of this protein over the substrate and differential osteogenic capacity within the construct. The osteogenic character of the produced cell sheets was further confirmed by Alizarin Red-S staining (Figure S2) that revealed an intense staining due to high calcium deposition.

### Confluent Layer of Osteoblastic-like Cells Derived from hBMSCs Act as a Remodeling and Structural Substrate for Other Cell Types

**Figure 4:**
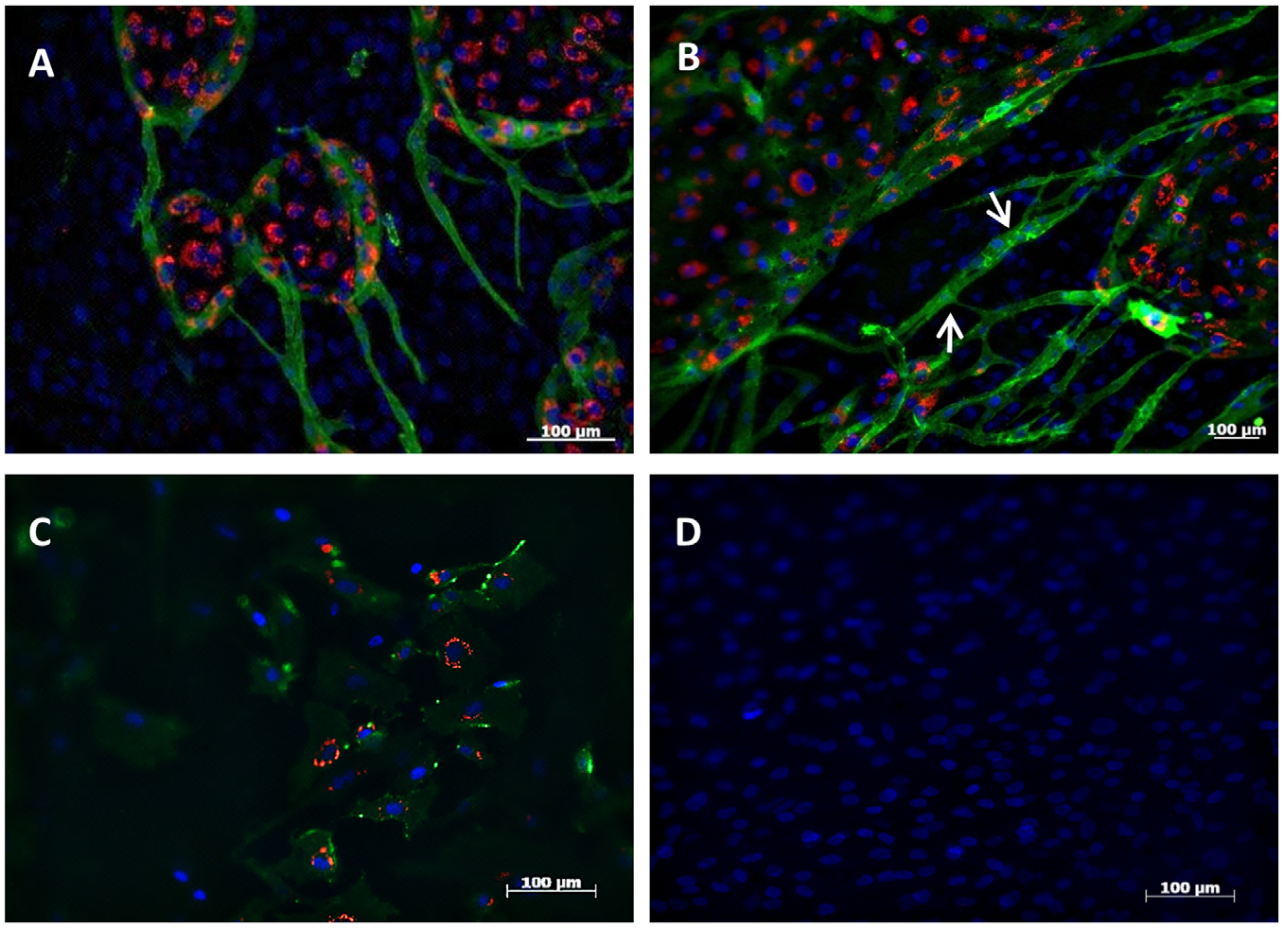
Immunocytochemistry for CD146 expression and Dil-AcLDL uptake by hBMSCs monocultures and co-cultures with perivascular-like (CD146^+^) cells and HUVECs, after 14 days of culture. (A,B) Co-cultures on hBMSCs-derived osteogenic cells showing endothelial colonies (red) and elongated perivascular-like (CD146^+^) cells (green) interacting with HUVECs and with them-self (Arrow). (C) Co-cultures of HUVECs (red) and perivascular-like (CD146^+^) cells (green) on plastic adherent conditions showing random organization. (D) Confluent layer of hBMSCs-derived osteogenic cells lacking the expression of CD146. DAPI (blue) was used as nuclear staining.

In addition to cell-cell interactions and signaling through PDGF and TGF on co-culture models of ECs and perivascular-like cells, the ECM serves as an adhesive support in which cells organize in multicellular structures and change their morphology and contractibility [35]. With this work a new co-culture model, composed by 3 different cell types, displaying characteristic cellular organization and distribution pattern is proposed. When cultured on the hBMSCs-derived osteogenic cells, HUVECs, incorporating DiL-AcLDL (red), organized in round colonies from 2 day onward while perivascular-like (CD146^+^) cells (green), displaying an elongated morphology (Figure 4A;B), were only observed from day 5 forward. The presence of a collagen-rich ECM produced by the osteoblastic cells appears to be the key regulator for this singular organization since co-cultures of HUVECs and perivascular-like (CD146^+^) cells on plastic adherent substrates did not exhibited the same organizational and morphological pattern (Figure 4C). The elongated shape of perivascular-like (CD146^+^) cells is also shown by vSMC *in vivo* and is strongly related to an effectively regulation of vessel distension and diameter [41], [42]. To determine whether the co-culture medium affected the expression of CD31 and CD146 on HUVECs and perivascular-like (CD146^+^) cells, monocultures of those cell types were maintained for 7 days in co-culture medium. The maintenance of the CD146 and CD31 phenotype (>98%) on HUVECs was confirmed. These results confirmed that the addition of osteo-inductive factors in the endothelial medium (M199) did not interfere with the endothelial phenotype neither with their survival. However, a slight decrease of CD146 expression was observed on perivascular-like (CD146^+^) cells, in comparison to the cultures in the presence of TGF-β1 (Figure 1C), both for the number of cells expressing the antigen (84%) as well as for the amount of antigen (data not shown). Also, morphological changes on perivascular-like (CD146^+^) cells were visible when cultured in the co-culture medium (Figure S3). Immunocytochemistry for CD146 on confluent hBMSCs after 14 days of induction with osteogenic medium revealed no connections mediated by CD146 adhesion molecule, nor Dil-AcLDL uptake (Figure 4D).

### Transplanted Pre-vascularized Osteogenic Cell Sheets Show Osteogenic and Angiogenic Potential

**Figure 5:**
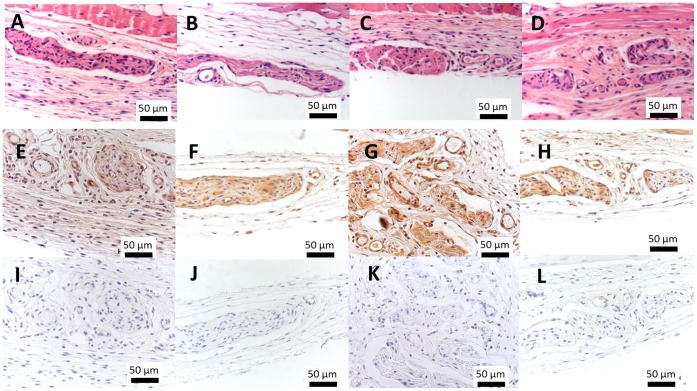
H&E staining and osteocalcin immunolocalization on explants retrieved 7 and 21 days after transplantation of cell sheet-based constructs. (A–D) H&E staining on (A;B )control and (C;D) experimental explants after 7 (A;C) and 21 days (B;D) of subcutaneous implantation showing their localization and morphology. (E–L) Immunolocalization of osteocalcin on (E;F) control and (G;H) experimental explants at 7 (E;G) and 21 (F;H) days of implantation revealing osteogenic commitment on both test conditions. (I–L) immunostaining negative control of respective E–H conditions.

Explants were histologically analyzed in order to infer about the osteogenic and angiogenic potential of the transplanted cell sheet-based constructs. Single osteogenic cell sheets were used as control condition for *in vivo* experiments. H&E staining revealed the presence of some perfused blood vessels around and inside transplanted cell sheets after 7 days of implantation, both on control and experimental conditions (Figure 5A–D). The osteogenic character of the retrieved samples was confirmed by the identification of osteocalcin (Figure 5E–H).

**Figure 6:**
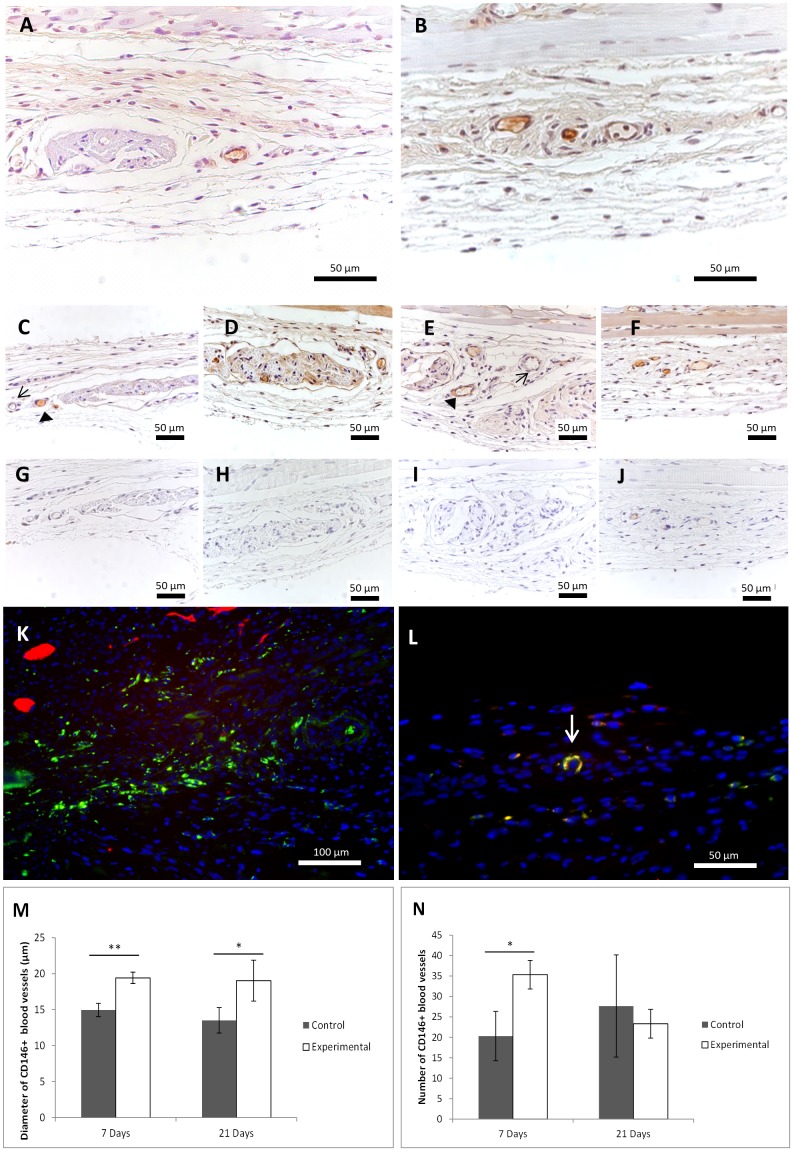
Angiogenic potential of the transplanted cell sheet-based constructs. Immunohistochemistry for (A;B) CD31 and (C–F) CD146 on (C;D) control and (E,F) experimental conditions at days 7 (C;E) and 21 (D;F) of implantation; (G–J) Immunostaining negative control of respective conditions. → negative blood vessels for CD146; ▸ positive blood vessel for CD146. (K) Human cells (green) detected using human-specific anti-mitochondria antibodies on the experimental condition 7 days after implantation. (L) Co-localization (yellow) of CD146 (red) and human-specific anti-mitochondria (green) revealed cellular assembling in a blood vessel-like structure (arrow) on the experimental condition 7 days after implantation. DAPI (blue) was used as nuclear staining. Representation of (M) the mean diameter and (N) the number of CD146 positive vessels present on control and experimental conditions at days 7 and 21 of implantation. *p≤0.05; **p≤0.01.

The contribution of the transplanted HUVECs for the development of new blood vessels, namely their integration in the vascular network within the explants, was confirmed by the presence of human CD31 positive cells on those vessels both at 7 and 21 days of implantation (Figure 6A;B). In what concerns the contribution of the perivascular-like (CD146^+^) cells, it is interesting to find a differential expression of CD146 antigen on the explants blood vessels both on experimental and control conditions (Figure 6C–F), as not all the vessels were labeled. In order to specifically identify human CD146^+^ cells, the co-localization of CD146 antigen and of human mitochondria was performed (Figure 6K;L). The transplanted human cells were identified within the tissue with osteogenic characteristics resulting from the cell sheet construct implantation (Figure 6K). Moreover, human cells co-expressing the CD146 surface marker, corresponding to perivascular-like (CD146^+^) cells and HUVECs, were also found after 7 days of implantation, as part of a vessel-like structure confirming the involvement of the transplanted cells in the formation of a new vascular network (Figure 6L). The diameter of CD146^+^ blood vessels, as well as the total number of new CD146 positive blood vessels formed were assessed both for experimental and control conditions (Figure 6M;N). Only the blood vessels formed between skin skeletal muscle and the mice connective tissue were considered. A significant increase of the diameter of CD146 stained blood vessels was observed, in comparison to the control, in the experimental condition after 7 (p≤0.01) and 21 (p≤0.05) days of transplantation. The same was observed regarding the total number of CD146 stained vessels although a significant difference (p≤0.05) was only observed for 7 days of implantation. The CD146 stained blood vessels on the experimental conditions maintained a size of approximately 20 µm from 7 to 21 days while in the control depicted an average diameter of 15 µm.

## Discussion

The main objective of this work was the development of a model combining osteogenic, endothelial and perivascular-like cells, as a strategy to enhance the vascularization of bone TE constructs. Considering that the pre-vascularization of tissue engineered substitutes constitutes a valuable approach to improve its survival after transplantation, we hypothesized that the incorporation of perivascular-like (CD146^+^) cells, directly interacting with endothelial cells, could further enhance that effect by promoting the maturation and stabilization of the newly formed vasculature. Thus, to test our hypothesis, we established a human cell-sheet model based on our previous works that showed that rat bone marrow-derived mesenchymal stem cells have the capacity to form osteogenic cell sheets and, in combination with HUVECs, lead to improved vascularized bone tissue formation [7], [43]. Due to distinct osteogenic differentiation patterns between rat and human MSCs [44] the two main features needed to produce a workable cell sheet-based construct, the secretion of ECM that has to be sufficient to allow cell sheet detachment and confer robustness, and the mineralization degree that cannot hinder its detachment (Figure S1), were optimized. Standard osteogenic conditions gave rise to a fragile monolayer of cells, involved by an ECM composed by collagen type-I and osteocalcin that was not sufficient to allow cell manipulation. Therefore we were able to compensate the deficient integrity of the osteogenic cell sheets by inducing ECM production through the supplementation of the osteogenic medium with ascorbic acid, known to stimulate proliferation rate and induce the secretion of ECM [45], at a concentration 3 times higher than the standard conditions. The higher contractibility degree of the cell sheets after detachment is not expected to have consequences for their application since the standard recovery procedures, involving the use of a PVDF membrane or gelatin coated manipulators, avoid that effect.

Extensive research concerning the role of pericytes in vasculature stabilization has been contributing for the discovery of possible new pericyte functions, including regulation of endothelial proliferation and differentiation, microvascular perfusion, permeability regulation through paracrine agents [46], [47] and regulation of epithelial proliferation and tissue regeneration [48]. The potential role of CD146^+^ cells, selected from bone marrow, to act as pericytes was proposed by Caplan [26]. According to Anfosso and colleges [49], CD146 can act as a signaling molecule in the dynamics of cytoskeleton rearrangement on HUVECs. In this work we showed the inducible characteristic of this molecule, both *in vivo* and *in vitro*, by demonstrating changes on the CD146 expression of perivascular-like (CD146^+^) cells and the consequent morphological variation observed under different *in vitro* conditions, such as culture media and the presence of TGB-β1, and the existence of CD146 negative and positive blood vessels *in vivo*.

To date, works regarding the study of cellular interactions between osteoblasts and endothelial cells in co-culture systems resulted in a significant collection of new data concerning the molecular intervenients [50]–[56]. However, as far as we know, the behavior of endothelial and perivascular-like (CD146^+^) cells, cultured together over a confluent layer of osteoblastic cells, was not documented before. In this work, the ability of the confluent layer of osteoblastic cells derived from hBMSCs to act as an organizational structure for endothelial and perivascular-like (CD146^+^) cells was shown. In the absence of the osteogenic substrate endothelial and perivascular-like (CD146^+^) cells were randomly distributed. When cultured on the osteoblastic cells endothelial cells organized themselves in colonies after two days. This organization has been previously shown [52] but others have also reported spontaneously self-assembly of endothelial cells in tubular-like structures when co-cultured with MSCs or osteoprogenitor cells on plastic culture surfaces [50], [51], [57] or as spheroids [54]. The micro and macrovascular character of the different endothelial cells that have been studied might contribute for the distinct observations however, a preliminary work developed in our lab with human dermal microvascular endothelial cells as the endothelial cells in the proposed co-culture system, lead us to confirm that other factors than the cell source have influence over the organization of the endothelial cells in *vitro* (*Data not shown).* It is well known that ECs behavior and functioning are under regulation of angiogenic cytokines, such as VEGF, however the appropriate ECM is equal or more important in terms of EC migration, survival and proliferation [35]. In fact, the importance of the ECM produced by osteoprogenitor cells for the storage and release of chemotactic factors [58], [59] as well as in the establishment of homotypic and heterotypic gap junctions for cell-to-cell communication on endothelial and osteoblastic cells co-culture models [60], [61] has been previously shown. TIMP-3, a matrix metalloproteinase highly expressed by pericytes [62], osteoblastic cells, mesenchymal stem cells and endothelial cells [63] has been implicated in the inhibition of endothelial tube formation [64], nevertheless, it is also strongly involved in promoting cell-cell junction formation and stability [35].

Interestingly, perivascular-like (CD146^+^) cells cultured on the osteogenic substrate also altered their “star-shape” morphology and reorganized them-selves in cord-like structures. In addition to the effect of the co-culture medium over perivascular-like (CD146^+^) cells morphology and of the osteogenic ECM, the cell-cell interactions and/or paracrine signaling are likely to contribute to the observed behavior. Interactions between HUVECs and CD146^+^ cells might be mediated by the release of some signaling molecules, such as PDGF-β, FGF and TGF-β, by HUVECs [36], [65]. The release of TGF-β by HUVECs is also a possible explanation for the maintenance of CD146 expression by perivascular-like (CD146^+^) cells in our co-culture, as shown by immunocytochemistry. According to Hirsch and D'Amore [66], TGF-β is released in a latent form and its activation is led by endothelial cells-pericytes contact, which corroborates the existence of causative cell-cell interactions in our model. PDGF-β and FGF are chemoattractants for vSMCs and mesenchymal derived cells [67]. Recently, Caplan and Correa [68] suggested a critical role for PDGF-β in the vascular-pericyte-MSC-osteoblast dynamics as a central connector between cellular components and osteoblast differentiation program. Although the nature of the mechanisms involved on this crosstalk were not addressed, the study of endothelial cells, pericytes and osteogenic ECM interactions are essential to understand how sprouting morphogenesis and vessel stabilization are regulated.

In addition to the *in vivo* osteogenic potential of the human cell sheet-based constructs, we also confirmed the integration of HUVECs within the host developed vasculature, thus demonstrating their active role in the angiogenic process, similarly to what was observed for rat cell sheet-based constructs [7]. Additionally the integration of human cells in host connective tissue and the organization of the transplanted human cells in vessel-like structures was confirmed. The specific contribution of perivascular-like (CD146^+^) cells was, however, not evident due the cross-reactivity of the CD146 antibody with mouse antigens and because CD146 is also expressed by endothelial cells. We can correlate the diameter of the CD146 positive blood vessels, higher on the experimental condition, with the presence of perivascular-like (CD146^+^) cells and with vessels maturation and stability. According to a theoretical model proposed by Pries *et al.* [69], increased vessel diameter and wall mass are needed to ensure stable vascular adaptation. Also, other work has demonstrated the development of larger caliber vessels *in vivo* when SMC were co-engrafted with EC into collagen gels, contributing to accelerate, stabilize and promote remodeling of tissue engineered microvessels [70]. This is considered indicative of vessel maturation and an effect of the recruitment of mural cells that induce vessel maturation by promoting the structural stabilization [71].

Furthermore, considering the importance of CD146 molecule for angiogenesis, the observed differential expression of CD146 on the developed vasculature seems to indicate that the expression of this molecule is related to increased blood vessels stability.

In summary, this work proved the capacity of hBMSCs to form osteogenic cell sheets and its role in modulating the assembly of two cell types intimately related to vasculature, the endothelial cells and perivascular-like (CD146^+^) cells. Moreover, the capacity of the human cell sheet-based construct to form vascularized osteogenic tissue *in vivo* with improved maturation and vessel stability reinforced that the proposed model constitutes a suitable starting element to further develop thicker cell dense constructs. This can be easily achieved by combining several layers of cells, including pre-vascularized cell sheets. In this context, the conception of using bone marrow cells as a source of perivascular-like and osteogenic-derived cells to create a co-culture model combining these with endothelial cells appears to be a useful strategy for the in vitro pre-vascularization of TE constructs and to improve its survival after implantation by promoting a stable and mature supplying vasculature.

## Materials and Methods

### Cell Isolation and Culture

Bone marrow aspirates were obtained after informed consent from patients undergoing hip replacement surgery, at Hospital da Prelada, Porto, Portugal. Human bone marrow-derived mesenchymal stem cells (hBMSCs) were isolated by gradient centrifugation as previously described [72] and maintained in complete α-MEM (Gibco, USA) supplemented with 2 ng/mL FGF-β (PeproTech, USA). Cells were used at passage between 2 and 3. Umbilical cords (UCs) obtained by caesarean section from healthy donors were provided by Hospital de S. Marcos, Braga, Portugal and delivered to the cell culture laboratory in transport buffer, containing 0.14 M NaCl, 0.004 MKCI and 0.011 M glucose in 0.001 M phosphate buffer at pH 7.4. The isolation of the human umbilical cord vein endothelial cells (HUVECs) was carried out as described in the literature by Jaffe and others [73], and cells were used up to passage 5. Biological samples were provided under a protocol approved by the Hospitals Ethical Committees and established with the 3B’s Research Group.

### Induction of CD146+ Phenotype

hBMSCs were cultured for 7 days in complete α-MEM supplemented with 1 ng/mL TGF-β1 (ebiosciences, USA). Culture medium was replaced twice during culture time and the differentiated CD146^+^ cells were used as perivascular-like (CD146^+^) cells to establish the co-cultures.

### Co-cultured Cell Sheets Fabrication

hBMSCS, at a density of 35.000 cells/cm^2^, were cultured on thermo-responsive dishes (Nunc, Danmark) for 7 days in complete α-MEM (Gibco, USA) supplemented with osteogenic differentiation factors, 10 mM β-Glycerophosphate (Sigma, USA), 150 µg/mL ascorbic acid (Sigma, USA) and 1×10^−8^ M dexamethasone (Sigma, USA). HUVECs and perivascular-like (CD146^+^) cells, in a total of 45.000 cells/cm^2^ and at a final ratio of 4∶1, were then seeded onto the confluent layer of hBMSCs and cultured in Medium 199 (Sigma, USA) supplemented with the osteogenic differentiation factors described above. After further 7 days, co-cultured cell sheets were retrieved by temperature decrease as previously described [74], fixed with 3.7% buffered formalin and paraffin embedded for histological characterization. For immunofluorescence characterization, the co-cultures were established on tissue culture polystyrene (TCPS) coverslips under the described conditions.

### Cell Sheet Stacking and Transplantation

Two types of cell sheets, single monocultured osteogenic cell sheets and co-cultured osteogenic cell sheets stacked with a second monocultured osteogenic cell sheets were transplanted, respectively as control and experimental conditions. To stack the two cell sheets, a poly(vinylidene diﬂuoride) (PVDF) membrane (Millipore, USA) with 2 cm of diameter was placed over an osteogenic cell sheet and incubated at RT for 15 minutes. After this time, the cell sheets spontaneously detached from thermoresponsive dishes and were attached to the membrane which allowed its manipulation and stacking over the co-cultures, still in TR dishes. The all construction was further incubated at room temperature for 15 minutes to allow the detachment of the co-cultured cell sheet from the TR dish and adhesiveness to the osteogenic one on top, forming a double cell sheet construct that combined two osteogenic cell sheets with HUVECs plus perivascular-like (CD146^+^) cells in between.

The transplantation of *in vitro* cultured cells sheets was carried out as previously reported [75]. Briefly, 5 weeks old male nude mice (Charles River, USA), n = 5 per condition and timepoint, were anesthetized with a mixture of ketamine (1.2 mg/mouse s.c., Imalgene® 1000, Merial, Lyon, France) and medetomidine (20 µg/mouse s.c., Domitor®, Orion Corp., Finland) prepared in physiological serum. After the confirmation of analgesia/anaesthesia, dorsal skin flap was cut opened using 3×3 cm cutting sides. Recovered cell sheets were placed on mouse subcutaneous dorsal flap and left to adhere to the connective tissue of dorsal skin for 5 minutes. After that time, the PVDF membrane was removed, and the skin flap was brought back to the original location and sutured.

### Cell Sheets Recovery

At each time point, animals were euthanized with an intracardiac overdose of anesthesia and implants were recovered for histological characterization by removing the skin flap following the suture marks. Skin flaps were then pinned on a piece of cork to prevent curling up and emerged in 3,7% formalin for 24 hours at 4°C before processing.

### Flow Cytometry

Flow cytometry was performed using mouse anti-human antibodies CD146 (unconjugated, abcam, UK), CD73 (PE-conjugated, BD biosciences, USA), CD90 (APC-conjugated, ebiosciences, USA) and CD105 (FITC-conjugated, AbD Serotec, UK). Experiments were performed using cells in different passages, from isolation day to P6, and obtained from different donors (n = 3). hBMSCs, perivascular-like (CD146^+^) cells and HUVECs were trypsinized, counted and resuspended in a 2% BSA (Sigma, Canada) solution in PBS (BSA/PBS) at a concentration of 2500 cells/µL. For indirect staining, cells were first incubated for 45 min at 4°C, protected from light, with CD146 antibody (1∶100). After a washing step with PBS, cells were incubated for 45 minutes, protected from light, at room temperature with AF488 conjugated secondary antibody (goat anti-mouse, Molecular probes, USA) at a concentration 1∶500. For direct staining, cells were incubated for 20 minutes at room temperature, protected from light, with the fluorescence-conjugated primary antibodies listed above. After a washing step, cells were resuspended in PBS and 20.000 counts were analyzed using a FACSCalibur flow cytometer (BD Biosciences) and the CELLQuest software V3.3.

### Immunofluorescence

Monocultures of perivascular-like (CD146^+^) cells and co-cultures established on the TCPS were incubated for 30 minutes with 3% BSA/PBS at room temperature. Then, cells were washed with PBS and incubated overnight at 4°C with mouse:anti-human CD146 antibody (1∶100) diluted in 3% BSA/PBS. Cells were then washed in PBS and incubated for 1 hour at room temperature with AF488 conjugated secondary antibody (goat:anti-mouse, Molecular probes, USA), diluted in 3%BSA/PBS to a 1∶500 concentration. Nuclei were counterstained with DAPI (3 µg/mL) by incubation for 30 minutes at room temperature. The co-cultures were previously incubated overnight at 37°C in humidified atmosphere and 5%CO_2_ with Dil-AcLDL (Molecular Probes, USA), at a final concentration 0.2 µg/mL and before fixation, to label HUVECs.

For the co-localization of human cells and CD146^+^ cells on the *in vivo* explants at different implantation periods, samples were treated for 5 minutes with alizarin red-S solution (2%) (Sigma, China) in order to quench auto-fluorescence. Mouse:anti-human CD146 and human specific anti-mitochondria (Milipore, USA) antibodies were incubated for 1 hour at room temperature followed by the incubation with AF488-conjugated (donkey anti-rabbit, Molecular probes, USA) and AF594-conjugated secondary antibodies (goat anti-mouse, Molecular probes, USA) at a concentration of 1∶500 for 1 hour at room temperature. DAPI was used as nuclei staining. Samples were analyzed using an Axioplan Imager Z1 fluorescence microscope (Zeiss, Germany) and images were acquired and treated with AxioVision V.4.8 software.

### Hematoxylin and Eosin Staining

Histological sections (5 µm) were first deparaffinized with Clear-rite3 (Thermo-Scientific, Germany) and rehydrated in graded alcohol series. Samples were stained with Mayer’s Haematoxylin (Sigma, USA), for 5 minutes, followed by a washing under tap water. Then samples were incubated in eosin solution (BioOptica, Italy) for 45 seconds, dehydrated in grade alcohol series and mounted.

### Immunohistochemistry

Immunohistochemistry was performed both for *in vitro* cultured cell sheets and *in vivo* explants using mouse:anti-human antibodies against CD146 (1∶100) (abcam, UK), CD31 (1∶40) (Dako, USA), Osteocalcin (1∶100) (AbD Serotec, UK) and type-I collagen (abcam, UK). Histological sections (5 µm) were first deparaffinized with Clear-rite3 (Thermo-Scientific, Germany) and rehydrated in graded alcohol series, followed by antigen retrieval with 10 mM sodium citrate buffer solution (pH 6) at 98°C for 20 minutes. For intracellular antigens, sections were treated with 0.5% Triton X100 (Sigma, USA) in PBS for 10 minutes. Unspecific binding was blocked using 2.5% normal horse serum (NHS) (Vector Labs, USA) for 30 minutes, flowed by 3 washing steps, 5 minutes each, with PSB-0.1%Tween20 (Sigma, Germany). Antibodies were diluted in 1.5% normal horse serum and incubated at room temperature for 1 hour. Sections were then washed 3 times in PBS-Tween, 5 minutes each, and the endogenous peroxidase activity was quenched with 0.3% in methanol (30 minutes). After a washing step, 3 times in PBS-Tween, 5 minutes each, sections were incubated for 30 minutes with biotinylated anti-mouse secondary antibody (Vector Labs, USA). After washing, sections were incubated for 30 minutes with R.T.U. vectastain elite ABC reagent (Vector Labs, USA) before HRP enzymatic activity was revealed with DAB (Vector Labs, USA). Nuclei were counterstained with Mayer’s hematoxylin followed by sections dehydration in graded ethanol series and mounting. In the case of *in vivo* samples, mouse Ig blocking reagent (M.O.M. kit, Vector Labs, USA) was applied for 1 hour before primary antibody incubation in order to reduce background. Samples were analyzed using the Axioplan Imager Z1 fluorescence microscope (Zeiss, Germany) and images were acquired and treated with AxioVision V.4 software.

### Statistical Analysis

Quantification of the mean diameter and the number of CD146 positive blood vessels formed on control and experimental groups *in vivo* was conducted in 3 animals per group and replicated in at least 6 sections per condition. The statistical analysis was performed using one-way ANOVA and results are considered statistically different for *P* values lower than 0.05.

## Supporting Information

Figure S1 **Representative flow cytometry analysis of CD73, CD90 and CD105 expression on hBMSCs.** (A;B) Expression of MSCs markers CD73, CD90 and CD105 on bone marrow mononuclear fraction at isolation day. (C;D) CD73, CD90 and CD105 expression on hBMSCs (P5) cultured in complete α-MEM; (E;F) CD73, CD90 and CD105 expression on hBMSCs (P5) cultured for 7 days in complete α-MEM supplemented with 1 ng/mL TGF-β1.
(TIF)

Figure S2 **Macroscopic view of hBMSCs cell sheets cultured in thermoresponsive dishes with osteogenic medium.** (A) Osteogenic cell sheet cultured for 14 days in osteogenic medium after detachment and contraction. (B) Osteogenic character of cell sheet after 21 days in culture with osteogenic medium reveal by Alizarin Red-S staining.
(TIF)

Figure S3 **Contrast phase microscopy of perivascular-like (CD146^+^) cells cultured for 7 days in Medium 199 supplemented with osteogenic factors.** Morphological chances were visible when compared with the same cells in culture with complete α-MEM or α-MEM supplemented with TGF-β1.
(TIF)
