# Supplementary figures and images for: Perivascular-Like Cells Contribute to the Stability of the Vascular Network of Osteogenic Tissue Formed from Cell Sheet-Based Constructs
Source: PLoS One. 2012 Jul 19;7(7):e41051. doi: 10.1371/journal.pone.0041051 (PMC3400580; doi:10.1371/journal.pone.0041051)

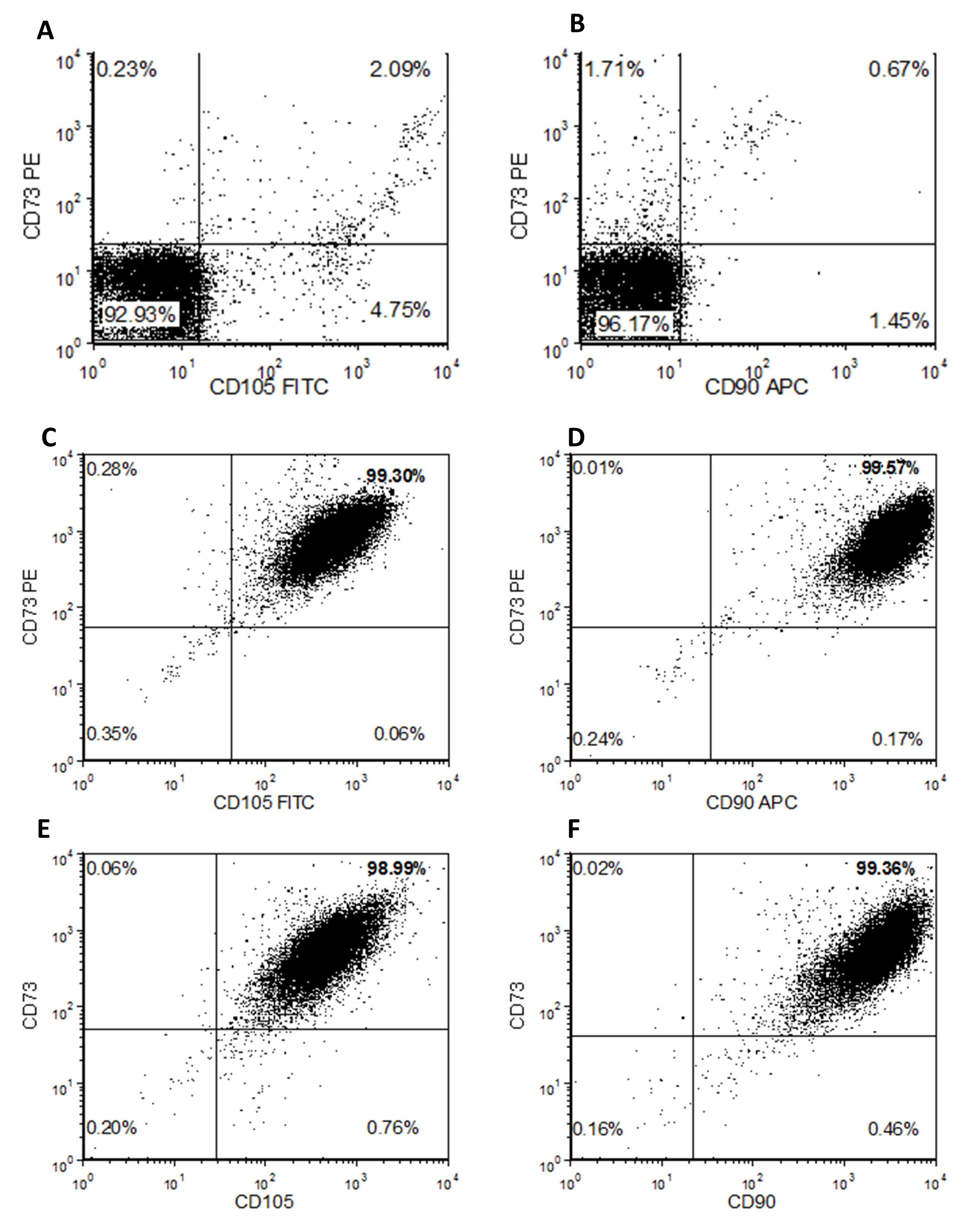

Supplement: Figure S1 — Representative flow cytometry analysis of CD73, CD90 and CD105 expression on hBMSCs. (A;B) Expression of MSCs markers CD73, CD90 and CD105 on bone marrow mononuclear fraction at isolation day. (C;D) CD73, CD90 and CD105 expression on hBMSCs (P5) cultured in complete α-MEM; (E;F) CD73, CD90 and CD105 expression on hBMSCs (P5) cultured for 7 days in complete α-MEM supplemented with 1 ng/mL TGF-β1. (TIF) [file pone.0041051.s001.tif]

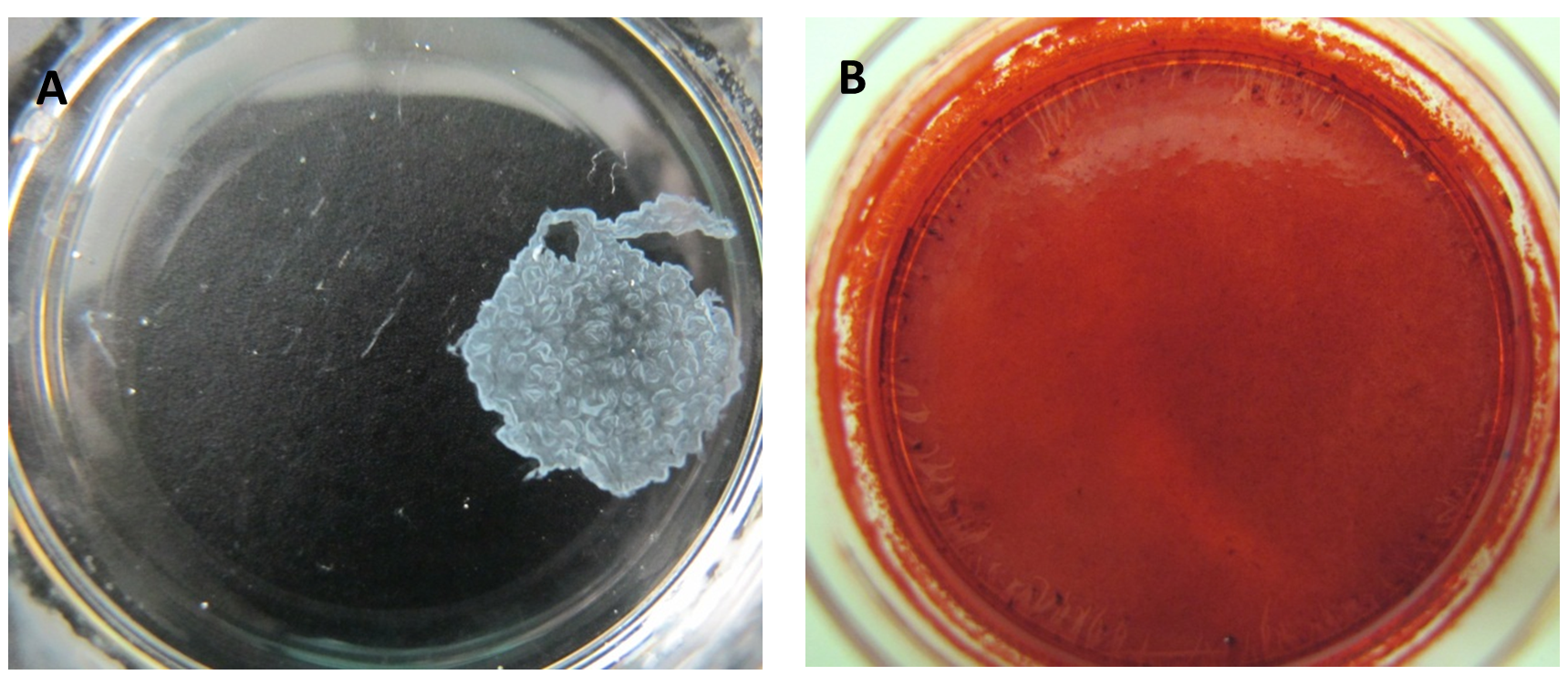

Supplement: Figure S2 — Macroscopic view of hBMSCs cell sheets cultured in thermoresponsive dishes with osteogenic medium. (A) Osteogenic cell sheet cultured for 14 days in osteogenic medium after detachment and contraction. (B) Osteogenic character of cell sheet after 21 days in culture with osteogenic medium reveal by Alizarin Red-S staining. (TIF) [file pone.0041051.s002.tif]

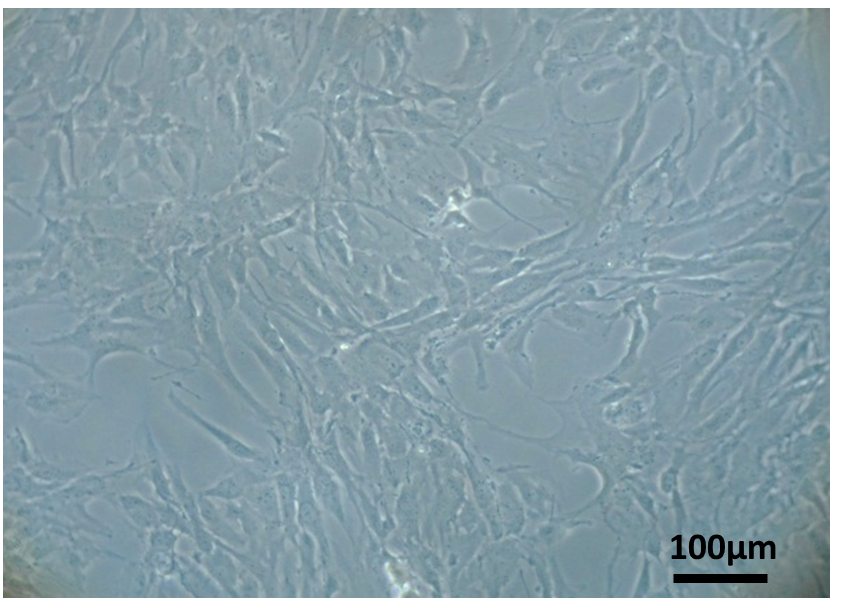

Supplement: Figure S3 — Contrast phase microscopy of perivascular-like (CD146+) cells cultured for 7 days in Medium 199 supplemented with osteogenic factors. Morphological chances were visible when compared with the same cells in culture with complete α-MEM or α-MEM supplemented with TGF-β1. (TIF) [file pone.0041051.s003.tif]
